# Hearing Aid Use at the Intersection of Race, Ethnicity, and Socioeconomic Status

**DOI:** 10.1001/jamahealthforum.2024.3854

**Published:** 2024-11-22

**Authors:** Sarah Bessen, Wuyang Zhang, Emmanuel E. Garcia Morales, Ellesse-Roselee L. Akré, Nicholas S. Reed

**Affiliations:** 1 Department of Otolaryngology-Head and Neck Surgery, Johns Hopkins University, Baltimore, Maryland; 2 Cochlear Center for Hearing and Public Health, Johns Hopkins University, Baltimore, Maryland; 3 Department of Epidemiology, Johns Hopkins Bloomberg School of Public Health, Baltimore, Maryland; 4 Department of Health Policy and Management, Johns Hopkins Bloomberg School of Public Health, Baltimore, Maryland; 5 Optimal Aging Institute, New York University Grossman School of Medicine, New York

## Abstract

This cross-sectional study examines self-reported uptake of hearing aids and devices among older adult participants in the National Health and Aging Trends Study.

## Introduction

Racial and ethnic inequalities are pervasive across hearing health care, including hearing aid use.^1^ There is a paucity of research on inequalities in hearing health care that consider the intersections of race, ethnicity, and socioeconomic status (SES).

## Methods

We conducted a cross-sectional analysis of adults aged 65 years or older with audiometric hearing loss using data from the 2022 National Health and Aging Trends Study (NHATS), a nationally representative sample of adults 65 years or older.^2,3^ In accordance with the Common Rule, this study was exempt from ethics review and informed consent requirement due to use of deidentified, publicly available secondary data. We followed the STROBE reporting guideline.

Primary exposures were self-reported race and ethnicity: Hispanic, non-Hispanic Black, non-Hispanic White, and other (American Indian or Alaska Native, Asian, Native Hawaiian or Other Pacific Islander, and other [specified by participant or proxy]) and annual household income based on the 2022 federal poverty level (low, middle, or high). The primary outcome was hearing aid use, which was defined by self-reported hearing aid or other hearing device use within the past month.

We estimated predicted adjusted percentages (PAPs) using weighted multivariable logistic regression models adjusted for demographic, socioeconomic, and health characteristics to examine the association between each exposure and the primary outcome. Race models were then stratified by income. Survey weights were applied to generate nationally representative estimates. Analyses were completed using Stata 18.0 (StataCorp LLC).

## Results

Among 3054 participants, 1442 (51.3%) were males and 1612 (48.7%) were females, with a mean (SD) age of 80.2 (6.8) years. A lower proportion of racially and ethnically minoritized participants reported hearing aid use compared with White participants (Black: 9.3% [62], Hispanic: 9.9% [45], other: 14.4% [17] vs White: 31.8% [735]) (Table 1). Regression models suggest that Black and Hispanic participants reported lower use of hearing aids compared with White participants (PAP, 16.6 [95% CI, 11.1-22.1], 13.7 [95% CI, 7.9-18.6], and 30.1 [95% CI, 27.7-32.6], respectively) (Table 2). When stratified by income, Black and Hispanic participants consistently had lower self-reported hearing aid use compared with White participants (low income: 6.8 [95% CI, 0.0-16.4], 5.3 [95% CI, 0.0-12.6], and 21.8 [95% CI, 12.3-31.3], respectively; middle income: 11.3 [95% CI, 3.4-19.2], and 16.4 [95% CI, 3.9-28.8], and 23.8 [95% CI, 16.8-30.8], respectively; high income: 19.3 [95% CI, 13.1-25.6], 17.5 [95% CI, 9.6-25.4], and 32.9 [95% CI, 29.0-36.8], respectively) (Table 2).

**Table 1.** Characteristics of National Health and Aging Trend Study Round 12 Participants by Race and Ethnicity

Characteristic	Participants, No. (%)					*P* value^a^
	Total (N = 3054)	Black (n = 530)	Hispanic (n = 342)	White (n = 2086)	Other (n = 96)^b^	
Age, mean (SD), y	80.2 (6.8)	79.9 (7.1)	78.0 (6.8)	80.6 (6.8)	79.5 (6.7)	<.001
Sex						
Female	1612 (48.7)	319 (55.8)	171 (53.7)	1078 (48.6)	44 (43.1)	.001
Male	1442 (51.3)	211 (44.2)	171 (53.7)	1008 (51.4)	52 (56.9)	
Marital status	1499 (58.7)	162 (36.2)	161 (51.7)	1117 (60.6)	59 (72.6)	<.001
Educational level						
<High school	547 (13.3)	162 (30.3)	182 (52.5)	184 (7.8)	19 (19)	<.001
High school diploma	750 (23.9)	138 (27.9)	51 (13.3)	546 (25.3)	15 (10.1)	
Some college	848 (28.4)	146 (28.6)	67 (18)	610 (29.6)	25 (23.3)	
≥College degree	909 (34.4)	84 (13.1)	42 (16.3)	746 (37.3)	37 (47.5)	
Household income level^c^						
Low	461 (11.5)	152 (30)	152 (42.1)	130 (6.2)	27 (26.8)	<.001
Middle	747 (21.7)	167 (28.2)	107 (28.9)	449 (20.2)	24 (27.2)	
High	1846 (66.8)	211 (41.8)	83 (29)	1507 (73.6)	45 (46)	
Medicaid	489 (12.5)	175 (35.9)	157 (45.1)	129 (6.7)	28 (25.7)	<.001
Tricare	186 (5.8)	34 (5.2)	13 (3.2)	128 (6)	11 (8.6)	.04
Residential region						
Northeast	483 (17.9)	72 (13.2)	31 (11.4)	372 (19.4)	8 (8.5)	<.001
Midwest	666 (23.3)	117 (22.6)	12 (2.3)	528 (25.6)	9 (17.7)	
South	1283 (38.5)	310 (57)	183 (49.9)	751 (35.8)	39 (37.7)	
West	622 (20.4)	31 (7.1)	116 (36.4)	435 (19.2)	40 (36.1)	
Metropolitan residence	2506 (80.4)	484 (88.4)	322 (92.4)	1616 (78.1)	84 (89.2)	<.001
No. of comorbidities						
0	418 (16.0)	39 (9.7)	44 (16.9)	323 (16.4)	12 (17.9)	<.001
1	935 (32.4)	149 (27.8)	89 (25.4)	674 (33.8)	23 (25)	
2	944 (29.9)	193 (32.9)	112 (28.7)	604 (29.5)	35 (36.5)	
≥3	757 (21.6)	149 (29.6)	97 (29)	485 (20.3)	26 (20.7)	
Hearing category						
Mild	1719 (62.1)	342 (74.4)	200 (66.1)	1122 (60.5)	55 (67.0)	<.001
Moderate or worse	1335 (37.9)	188 (25.6)	142 (33.9)	964 (39.5)	41 (33.0)	
Hearing aid use	859 (27.8)	62 (9.3)	45 (9.9)	735 (31.8)	17 (14.4)	<.001

^a^ Two-sided *P* < .05 indicated statistical significance.

^b^ Other category includes American Indian or Alaska Native, Asian, Native Hawaiian or Other Pacific Islander, and other (specified by participant or proxy).

^c^ Income categories were annual household income according to the 2022 federal poverty level (FPL), including low (less than the FPL), middle (between 100% and 200% of the FPL), and high (greater than 200% of the FPL). The complex analytical weights used by the National Health and Aging Trend Study were applied to all analyses.

**Table 2.** Self-Reported Hearing Aid Use Among National Health and Aging Trends Study Round 12 Participants^a^

Race and ethnicity^b^	Full sample (N = 3054)		Low income (n = 482)^c^		Middle income (n = 737)^c^		High income (n = 1835)^c^	
	PAP (95% CI)^d^	*P* value^e^	PAP (95% CI)^d^	*P* value^e^	PAP (95% CI)^d^	*P* value^e^	PAP (95% CI)^d^	*P* value^e^
Black	16.6 (11.1-22.1)	<.001	6.8 (0.0-16.4)	.17	11.3 (3.4-19.2)	.007	19.3 (13.1-25.6)	<.001
Hispanic	13.7 (7.9-18.6)	<.001	5.3 (0.0-12.6)	.14	16.4 (3.9-28.8)	.01	17.5 (9.6-25.4)	<.001
White	30.1 (27.7-32.6)	<.001	21.8 (12.3-31.3)	<.001	23.8 (16.8-30.8)	<.001	32.9 (29.0-36.8)	<.001
Other^f^	11.5 (2.2-20.8)	<.001	6.9 (0.0-16.7)	.16	8.6 (0.0-20.4)	.14	15.2 (0.0-30.0)	.045

Abbreviation: PAP, predicted adjusted percentage.

^a^ All models were adjusted for age, male sex, marital status (married vs single/widowed/divorced/never married), educational level (<high school, high school diploma, some college, and ≥college), Medicaid, Tricare, residential region (Northwest, Midwest, South, and West), metropolitan residence, number of chronic comorbidities (0, 1, 2, and ≥3; comorbid conditions include hypertension, diabetes, heart disease, lung disease, cancer, stroke, and myocardial infarction), and hearing category (mild vs moderate or worse). For the low-income subgroup analysis, Tricare was excluded from the list of covariates due to Tricare perfectly predicting the outcome for some individuals. The complex analytical weights used by the National Health and Aging Trend Study were applied to all analyses.

^b^ Race and ethnicity were self-reported by participants.

^c^ Income categories were annual household income according to the 2022 federal poverty level (FPL), including low (below the FPL), middle (between 100% and 200% of the FPL), and high (greater than 200% of the FPL).

^d^ For the full sample analysis, the PAP for each income level was 22.8 (95% CI, 13.5- 32.13) for low income, 22.0 (95% CI, 17.4-26.6) for middle income, and 30.0 (95% CI, 26.9-33.1) for high income.

^e^ Two-sided *P* < .05 indicated statistical significance.

^f^ Other category includes American Indian or Alaska Native, Asian, Native Hawaiian or Other Pacific Islander, and other (specified by participant or proxy).

## Discussion

In a nationally representative sample of US older adults, lower percentages of Black and Hispanic NHATS participants reported using a hearing aid compared with White participants. Similarly, Black and Hispanic participants across income levels had significantly lower self-reported hearing aid uptake than their White counterparts.

Given the persistence of racial gaps in hearing aid use after controlling for income and other socioeconomic factors, more nuanced investigations are needed into the implications of race, ethnicity, and SES for hearing aid use to elucidate barriers to uptake. While such racial gaps may be associated with economic factors and barriers to routine health care access, they are not fully explained by traditional indicators of SES, including income. Additionally, standard markers of high SES may not adequately characterize or quantify the relationships between SES and race.^4^ Future studies should consider factors such as economic hardship, wealth, access to bank credit, occupational or economic returns on education, and purchasing power.^4,5^

Study limitations include use of aggregated race and ethnicity categories that may not represent the substantial variability in racial and ethnic minoritized communities and inability to account for immigration status, experience with and role of systemic and interpersonal discrimination and racism, and other social and environmental factors affecting hearing aid use. Future research is needed to explain the downstream implications of these factors. Nevertheless, these findings emphasize that racial and ethnic disparities in hearing aid use would likely persist without more targeted efforts to increase access and reduce inequalities for populations at risk for hearing loss.

## Notes

Supplement.
Data Sharing Statement
