# Supplementary material for: Hearing Aid Use at the Intersection of Race, Ethnicity, and Socioeconomic Status
Source: JAMA Health Forum. 2024 Nov 22;5(11):e243854. doi: 10.1001/jamahealthforum.2024.3854 (PMC11584920; doi:10.1001/jamahealthforum.2024.3854)
Supplement: Supplement. — Data Sharing Statement [file jamahealthforum-e243854-s001.pdf]

Data Sharing Statement

Bessen. Hearing Aid Use at the Intersection of Race, Ethnicity, and Socioeconomic Status. *JAMA Health Forum*. Published November 22, 2024. doi:10.1001/jamahealthforum.2024.3854

Data
Data available: Yes
Data types: Data (not involving human participants)
How to access data: The data that support the findings of this study are available from the corresponding author upon reasonable request.
When available: With publication

Supporting Documents
Document types: None

Additional Information
Who can access the data: The data that support the findings of this study are available from the corresponding author upon reasonable request.
Types of analyses: For any purpose
Mechanisms of data availability: With investigator support
